# CRYSTALP2: sequence-based protein crystallization propensity prediction

**DOI:** 10.1186/1472-6807-9-50

**Published:** 2009-07-31

**Authors:** Lukasz Kurgan, Ali A Razib, Sara Aghakhani, Scott Dick, Marcin Mizianty, Samad Jahandideh

**Affiliations:** 1 Department of Electrical and Computer Engineering, University of Alberta, Edmonton, Alberta, Canada; 2 Department of Medical Physics, Shiraz University of Medical Sciences, Shiraz, Iran

## Abstract

**Background:**

Current protocols yield crystals for <30% of known proteins, indicating that automatically identifying crystallizable proteins may improve high-throughput structural genomics efforts. We introduce CRYSTALP2, a kernel-based method that predicts the propensity of a given protein sequence to produce diffraction-quality crystals. This method utilizes the composition and collocation of amino acids, isoelectric point, and hydrophobicity, as estimated from the primary sequence, to generate predictions. CRYSTALP2 extends its predecessor, CRYSTALP, by enabling predictions for sequences of unrestricted size and provides improved prediction quality.

**Results:**

A significant majority of the collocations used by CRYSTALP2 include residues with high conformational entropy, or low entropy and high potential to mediate crystal contacts; notably, such residues are utilized by surface entropy reduction methods. We show that the collocations provide complementary information to the hydrophobicity and isoelectric point. Tests on four datasets show that CRYSTALP2 outperforms several existing sequence-based predictors (CRYSTALP, OB-score, and SECRET). CRYSTALP2's accuracy, MCC, and AROC range between 69.3 and 77.5%, 0.39 and 0.55, and 0.72 and 0.79, respectively. Our predictions are similar in quality and are complementary to the predictions of the most recent ParCrys and XtalPred methods. Our results also suggest that, as work in protein crystallization continues (thereby enlarging the population of proteins with known crystallization propensities), the prediction quality of the CRYSTALP2 method should increase. The prediction model and the datasets used in this contribution can be downloaded from .

**Conclusion:**

CRYSTALP2 provides relatively accurate crystallization propensity predictions for a given protein chain that either outperform or complement the existing approaches. The proposed method can be used to support current efforts towards improving the success rate in obtaining diffraction-quality crystals.

## Background

Structural genomics is a word-wide initiative aimed at producing a comprehensive mapping of the protein structure space [1]. The resulting knowledge of the tertiary structure of proteins will be vitally important for understanding and manipulating the biochemical and cellular functions of a given protein. This is an important step in rational drug design [2] and provides valuable insights into important diseases [3]. There are several different ways to obtain the structure including X-ray diffraction, electron microscopy, and NMR. Although a majority of protein structures are obtained using the first method, the two latter approaches play a strong complementary role for some protein types, such as membrane proteins [4-6]. One of the main challenges the structural genomics initiative faces it that only about 2–10% of protein targets pursued yield high-resolution protein structures [7]. Several strategies have been proposed to improve the success rate, including obtaining one representative structure per protein family and working with multiple orthologues [8-11]. One of the most important bottlenecks in acquiring the structures is obtaining diffraction-quality crystals [12-14]. At the same time, crystallization is characterized by a significant rate of attrition and is among the most complex and least understood problems in structural biology [10]. Current protocols yield crystals for approximately 30% of the input proteins and well-diffracting crystals for an even smaller fraction [10]. This motivated the development of models that can be used to either support or directly predict protein crystallization [15]. For instance, the isoelectric point (pI) calculated from a primary sequence was used in a method that suggests optimal pH ranges for crystallization screening [16,17]. Several other investigations suggest that features derived from protein sequences can be used for predicting crystallization propensity [18,19]. To this end, a few in-silico methods that predict crystallization propensity using the primary sequence as the input have recently been developed. They include SECRET [20], OB-Score [21], CRYSTALP [22], and most recently ParCrys [23]. SECRET and CRYSTALP accept only sequences between 46 and 200 amino acids (AAs) in length. Although OB-score does not impose a limit on sequence size, it considers only two predictive features (pI and hydrophobicity), which limits the quality of its predictions. The ParCrys method extends OB-score by using a kernel-based classification algorithm and adding the composition vector of several amino acids (including Ser, Cys, Gly, Phe, Tyr, and Met) to the set of predictive features. All of these methods are built using black-box classification models, which are inductively learned from a set of protein chains, all annotated as crystallizable and noncrystallizable. By contrast, the XtalPred method [24] is a white-box approach that combines probabilities of successful crystallization calculated from several protein features. This method, which was developed based on experiences at the Joint Center for Structural Genomics, strives to mimic the work performed by structural biologists. XtalPred compares nine biochemical and biophysical features of an input protein with probability distributions estimated from data from the TargetDB database [25]. These features include protein length, molecular mass, Gravy and instability indices, extinction coefficient, isoelectric point, content of Cys, Met, Trp, Tyr, and Phe residues, insertions in the alignment compared to homologues in a non-redundant database of protein sequences, predicted secondary structure, predicted disordered, low-complexity and coiled-coil regions, and predicted transmembrane helices and signal peptides. The individual probabilities are combined into a single crystallization score which is used to assign one of five crystallization classes: optimal, suboptimal, average, difficult, and very difficult. The XtalPred provides a good benchmark for comparison since it uses a sophisticated sequence analysis (including several predictions) and models the routine "manual" work of structural biologists.

In the current article, we extend the CRYSTALP method to improve the quality of the predictions and to remove the sequence size restriction. When compared with CRYSTALP, the proposed CRYSTALP2 method uses new predictive features that are based on the collocation of amino acids in the sequence [22,26-29], includes information about pI and hydrophobicity, and applies a kernel-based classifier. Our goal is to provide a relatively simple method, i.e., we do not use sophisticated sequence analysis. We expect that our method will thus be complementary to current methods including XtalPred and ParCrys. We also note that many studies have shown that sequence-based prediction approaches, which may address a variety of structural and functional properties of proteins, provide useful information and insights for both basic research and drug design and hence are widely welcomed by the scientific community [30-34].

## Methods

Our methodology consists of two steps: (1) the protein sequence is converted into a fixed size feature vector, and (2) the feature values are entered into the classification model to predict the protein class (crystallizable/noncrystallizable). We followed the same design procedure as in [20,22] and our evaluation follows [20,22,23].

### Datasets

The design of the proposed method is based on a dataset of 418 proteins (hereafter D418) that includes 192 noncrystallizable and 226 crystallizable chains, which was introduced in [20]. Following the approach taken to design and test SECRET [20] and CRYSTALP [22], the design is based on tenfold cross-validation of the D418 dataset. We compare our out-of-sample predictions on D418 with SECRET and CRYSTALP. We also employ three datasets that were recently introduced in [23] and a new test dataset introduced in this contribution to compare CRYSTALP2 with CRYSTALP, SECRET, OB-Score, ParCrys and XtalPred. These four datasets are drawn from the TargetDB [25] and PepcDB  databases by applying procedures established in [23]. We use the FEAT dataset (composed of 1456 sequences, 728 crystallizable and 728 non-crystallizable) as the training dataset, while the TEST and TEST-RL datasets, composed of 144 (72 crystallizable and 72 non-crystallizable) and 86 (43 crystallizable and 43 non-crystallizable) sequences, respectively, are used as out-of-sample test sets. The sequences in the test datasets were made nonredundant (using CD-HIT [35] in the case of D418 and using AMPS [36] in the case of TEST and TEST-RL) to avoid any bias towards similar proteins and to assure independence between training and test data. The D418 and TEST-RL datasets include chains varying between 46 and 200 residues in length, while the FEAT and TEST dataset include chains of unrestricted length (minimum 42 and maximum 1169 residues). This experimental design is consistent with that in [23]. We also introduce a new test dataset of 2000 proteins (hereafter TEST-NEW), which is used to assess the quality of predictions for recently considered targets; we note that the FEAT, TEST and REST-RL datasets are based on proteins deposited before April 2007. This dataset simulates a large scale application of the proposed method, and was also developed following the procedure in [23]. The crystallizable proteins were extracted from sequences deposited in TargetDB. We selected the last 1000 depositions as of December 31, 2008 that are annotated as having "Diffraction-quality Crystals", and are not annotated with "In PDB" in the "Status" field. The resulting set includes proteins deposited between July 2006 and December 2008. The non-crystallizable sequences, which correspond to the actual construct sequences used, were extracted from the trial sequences stored in PepcDB. Sequences that are annotated as "work stopped" in the "Status" field and "Cloned" but not including an indicator of crystallization (e.g. "Crystals") in the "Status History" field were included in the set. Among these targets we removed DNA sequences, sequences which were annotated as "test target" and sequences for which "stopDetails" included "duplicate target found". As in the case of crystallizable chains, the remaining chains were filtered to select the last 1000 depositions as of December 31, 2008. The selected 2000 sequences were also processed to remove the N-terminal hexaHis tag (MGHHHHHHSH) and LEHHHHHH tag at the C-terminus, which are introduced to ease the purification; the same was done in [23]. Finally, we removed duplicate sequences and, as a result, the selected 2000 protein chains are nonredundant. Our results on this dataset are compared with the predictions of the ParCrys and XtalPred methods.

### Feature generation

The *Composition vector *was previously used to predict crystallization propensity [20,22,23]. Given 20 AAs (*A*, *C*,..., *W*, *Y*), ordered lexicographically, denoted as *AA*_1_, *AA*_2_,..., *AA*_19_, and *AA*_20_, and the number of occurrences of *AA*_i _in the sequence (denoted *n*_i_), the composition vector is defined as



where *k *is the length of the sequence.

The *amino-acid collocation vector *was first used in [22] and it is defined as the number of occurrences of two or more amino acids that are separated by gaps, i.e., amino acids of any type. CRYSTALP [22] employed a collocation vector for two amino acids (collocated dipeptides) that are separated by up to four gaps, i.e., *AA*_i_*AA*_j_, *AA*_i_-*AA*_j_, *AA*_i_--*AA*_j_, *AA*_i_---*AA*_j_, and *AA*_i_---*AA*_j_, where *AA*_i_*AA*_j _is a dipeptide, *AA*_i_-*AA*_j _is the same dipeptide separated by one amino acid of any type (denoted by -), etc. This yields 5*400 = 2000 collocation features. For CRYSTALP2 we also consider collocated tripeptides, which include 8000 tripeptides *AA*_i_*AA*_j_*AA*_k_, and 24000 tripeptides with single gaps, *AA*_i_*AA*_j_-*AA*_k_, *AA*_i_-*AA*_j_*AA*_k_, and *AA*_i_-*AA*_j_-*AA*_k_. In contrast to CRYSTALP, the number of occurrences for all collocated di- and tripeptides are normalized by the sequence length to allow predictions for sequences of unrestricted size. We note that local neighborhood information in the protein chain was also utilized in a recent method for design of crystallizable protein variants [37].

We also used pI and hydrophobicity as features. pI was used in OB-score [21], ParCrys [23] and XtalPred [24], and is strongly related to the efficiency of crystallization screening [16,17]. The pI values were computed using the ExPASy server [38] based on pK values of amino acids described in [39]. Sequence-based hydrophobicity was also used in [21,23]. As in [23], the hydrophobicity was calculated as the sum of Goldmann-Engleman-Steiz (GES) hydrophobicity values [40] for all residues, divided by the sequence length. The total number of features computed is 34,022.

### Feature selection

Since the initial feature set is relatively large, a feature selection method was used to reduce the number of features and to identify the most useful ones. We employed the correlation-based feature subset selection method (CFSS) [41], previously used to design CRYSTALP [22]. CFSS evaluates the value of a subset of features by considering the individual predictive capability of each feature along with the degree of redundancy between the features. The search strategy employed in feature selection was best first search. This search method explores the space of attribute subsets by using greedy hill-climbing with backtracking. Feature selection was performed using 10-fold-cross validation on the D418 dataset to avoid overfitting, and features that were deemed significant by CFSS in at least 1 fold were selected. Due to the large dimensionality of the initial feature set, feature selection was performed in two steps. First, we selected the best performing features from the composition and collocated dipeptides features (these were used in CRYSTALP), tripeptides, and collocated tripeptides. This resulted in the selection of 1103 features, i.e., 2 features from the composition vector, 94 from the collocated dipeptides, 250 from the tripeptides, 757 from the collocated tripeptides, pI, and hydrophobicity. These features were merged together and the feature selection was repeated. This resulted in a final set of 88 features, which are summarized in Table 1. We observe that only 15 of the selected features were also used by CRYSTALP; this is due to the normalization of the feature values and inclusion of new features in the proposed CRYSTALP2.

**Table 1:** Selected set of features.

**Composition**		**Collocated dipeptides**	**Collocated tripeptides**	**Other**
features	L, Y	DL, ES, GL, HH, IR, LF, LS, PP, QG, QM, RI, SS, SV, WC, WM, WV, WW, YI, YT, C-A, D-L, H-G, H-H, H-R, I-R, L-E, Q-L, R-S, T-K, T-S, T-T, D--M, F--S, H--C, H--H, K--W, L--N, S--L, T--G, W--W, Y--N, E---Q, E---S, F---T, G---H, L---D, L---L, Q---C, R---D, V---Y, Y---I, C----E, C----H, C----S, E----F, E----Q, G----R, I----E, L----L, M----V, M----Y, S----H, V----T, W----H, W----M	EFV, IVV, TKV, F-TK, K-TV, M-DS, P-PE, Q-QQ, R-PS, DP-V, LR-F, MG-S, SA-D, VT-G, YV-E, F-E-F, K-I-R, N-P-G, S-T-S	pI, average hydropho-bicity

# features	2	65	19	2

The underscored features in the "Collocated dipeptides" column indicate features that were also used in CRYSTALP.

### Classifier

The SECRET and ParCrys methods employ kernel-based classifiers as their prediction models. SECRET uses Support Vector Machines with Gaussian kernels, while ParCrys employs the Parzen window density estimator. We use another kernel-based technique, the normalized Gaussian radial basis function (RBF) network, which is a neural network with a hidden layer based on the non-linear Gaussian kernel function. In contrast to classical RBF networks [42], the normalized RBF (NRBF) networks have been shown to improve generalization, which leads to better performance on unseen test data [43]. We utilized the NRBF implementation in WEKA [44], in which the RBF functions are computed using the k-means clustering algorithm, i.e., symmetric multivariate Gaussians are fitted to the data for each k-means generated cluster, and the classification is based on logistic regression. This classifier requires the number of clusters, the width of the Gaussian kernel, and the ridge value for the logistic regression to be specified as training parameters. The number of clusters equals 2, which is the number of classes (prediction outcomes) in our problem. The other two parameters were selected based on a grid search using tenfold cross-validation tests on the D418 dataset. The best classification accuracy was obtained for a ridge value of 140 and kernel width 2.0. We note that each prediction generated by CRYSTALP2 is associated with a confidence score, defined as the difference between the probabilities of the two outcomes. The NRBF network generates a probability that a given input chain is predicted as crystallizable and as non-crystallizable. CRYSTALP2 predicts that a diffraction-quality crystal can be obtained when the confidence for this class is greater than that for the non-crystallizable class.

## Results and discussion

### Comparison with competing methods

**Figure 1:**
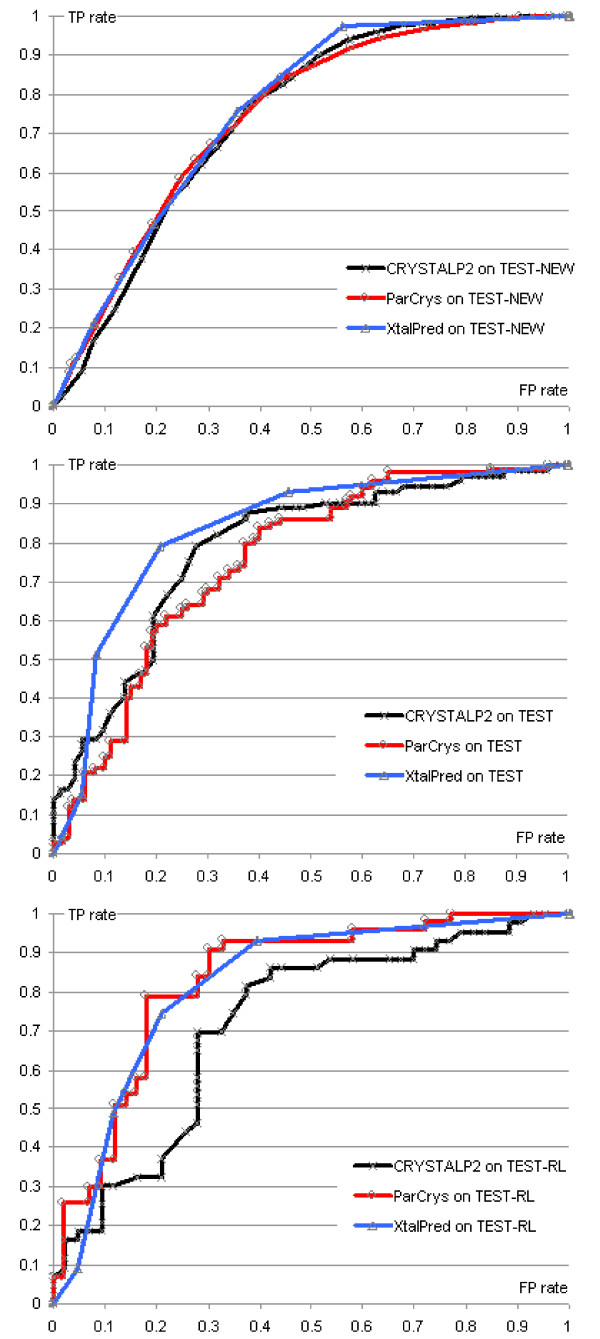
**ROC curves for ParCrys, XtalPred, and CRYSTALP2 on the TEST-NEW (top panel), TEST (middle panel) and TEST-RL (bottom panel) datasets**.

The CRYSTALP2 method was compared with SECRET, CRYSTALP, OB-Score, ParCrys, and XtalPred methods using two tests: the cross validation test on the D418 dataset, and a test in which the model was trained on the FEAT dataset and tested on the TEST, TEST-RL and TEST-NEW datasets. These tests mimic the testing procedures in [22,23]. We report the accuracy, Matthews's correlation coefficient (MCC), and area under the ROC curve (AROC) in Table 2. The ROC curve represents the relationship between the true positive (TP) and false positive (FP) rates; it is generated by establishing a threshold on the confidence scores from the predictors and then varying the threshold values. This allows the analyst to compare prediction qualities under different TP or FP rates, which is important when the analyst must consider different costs for Type I and Type II errors (i.e. is it more costly to wrongly abandon a crystallization attempt or to proceed with the attempt when it cannot succeed?) Results on the D418, TEST and TEST-RL datasets for the SECRET method were taken from [20] and [23]; the results for CRYSTALP, ParCrys, and OB-Score were taken from [22] and [23], and the XtalPred predictions were computed using the web server at [45]. The targets for which XtalPred generated optimal, suboptimal and average outputs were assumed to be crystallizable, while the remaining two classes (difficult and very difficult) were assumed to correspond to non-crystallizable chains. This assignment results in the best prediction quality. The results corresponding to other assignments are provided in the ROC curves, i.e. each point in the ROC curves of XtalPred that are shown in Figure 1 corresponds to one of potential assignments. ParCrys-W refers to predictions obtained by training the ParCrys method on a different dataset having an uneven number of crystallizable and non-crystallizable chains [23]. The TEST dataset includes sequences of unrestricted size, and thus only results for ParCrys, OB-score, and CRYSTALP2 are reported. The results on the TEST-NEW dataset for ParCrys and XtalPred were computed using web servers at  and , respectively.

**Table 2:** Comparison of prediction quality measured via accuracy, MCC and AROC between the proposed and five competing methods.

**Dataset**	**Method**	**Accuracy**	**MCC**	**AROC^6^**
D418^1^	SECRET	70.0	0.34	N/A
	CRYSTALP	77.5	0.55	N/A
	CRYSTALP2	77.5	0.55	N/A

TEST-RL^2^	CRYSTALP	46.5	-0.07	N/A
	SECRET	58.1	0.16	0.58
	ParCrys-W	67.4	0.38	0.84
	OB-Score	69.8	0.40	0.71
	ParCrys	79.1	0.58	0.84
	XtalPred^4^	76.7	0.54	0.82
	CRYSTALP2	69.8	0.40	0.72

TEST^2^	OB-Score	64.6	0.32	0.68
	ParCrys-W	68.0	0.37	0.75
	ParCrys	71.5	0.45	0.75
	XtalPred^4^	79.2	0.58	0.83
	CRYSTALP2	75.7	0.52	0.79

TEST-NEW	ParCrys^3^	70.6	0.43	0.75
	XtalPred^4^	70.0	0.40	0.76
	CRYSTALP2^5^	69.3	0.39	0.74

The AROC values for ParCrys, OB-Score and SECRET were taken from [23].

^1 ^Results based on tenfold cross-validation test on the D418 dataset

^2 ^Results based on training the classification model on FEAT dataset and testing on TEST-RL or TEST datasets, respectively

^3 ^Results based the ParCrys server at

^4 ^Results based the XtalPred server at

^5 ^Results based on training the classification model on FEAT dataset and testing on TEST-NEW datasets, respectively

^6 ^N/A means that the corresponding results was not reported and cannot be duplicated or computed

Table 2 shows that CRYSTALP2 provides an improvement over CRYSTALP. While both methods show the same quality on the D412 dataset, CRYSTALP performs relatively poorly on the TEST-RL dataset. This is likely due to the input features not being normalized in this method; the TEST-RL set has a different distribution of protein chain sizes than the D418 set. We observe that CRYSTALP2 obtains MCC = 0.4 on this test set, which is similar to the result of OB-Score and worse only than the results of ParCrys and XtalPred. At the same time, the proposed method outperforms all competing methods except XtalPred on the TEST set, which is larger than the TEST-RL dataset and contains chains of unrestricted size. The tests on the largest TEST-NEW dataset indicate that the three top performing methods, ParCrys, XtalPred and CRYSTALP2, provide similar performance with accuracy of about 70%, and MCC and AROC around 0.4 and 0.75, respectively.

The ROC curves in Figure 1 were generated for the three best performing methods (CRYSTALP2, ParCrys, and XtalPred) on the TEST, TEST-RL and TEST-NEW datasets to facilitate a more detailed comparison. We observe that for the TEST dataset CRYSTALP2 outperforms ParCrys for low and mid-range values of FP rate (when a relatively low number of chains is incorrectly classified as crystallizable), while ParCrys generates slightly higher TP rates for FP rate > 0.6. CRYSTALP2 would thus be more appropriate than ParCrys when the cost of incorrectly classifying a chain as crystallizable is significant. XtalPred is shown to generally outperform both ParCrys and CRYSTALP2 on this dataset. In the case of the TEST-RL dataset ParCrys and XtalPred are shown to provide favorable prediction quality when compared with CRYSTALP2. Finally, the ROC curves on the largest TEST-NEW dataset show that the three methods are characterized by similar performance across the entire range of the FP and TP rates. Overall, although XtalPred seems to provide good performance on all three datasets, we observe that there is no clear cut winner and that all three methods provide relatively comparable prediction quality.

This finding of similar performance prompted an investigation into the complementarity of the top three prediction methods. We compared predictions of CRYSTALP2 with the predictions of XtalPred and ParCrys by grouping them into four categories: 1) predictions that were correct for both CRYSTALP2 and XtalPred (or ParCrys); 2) predictions that were correct for CRYSTALP2 and incorrect for XtalPred (or ParCrys); 3) predictions that were incorrect for CRYSTALP2 and correct for XtalPred (or ParCrys); and 4) predictions that were incorrect for both CRYSTALP2 and XtalPred (or ParCrys). The results are shown in Table 3; we note that we could not duplicate the results of ParCrys in [23] by using the web server and thus we could not include a comparison with this method on the TEST and TEST-RL datasets in Table 3. The scores from categories (1) and (4) show overlapping results, while the second and third categories represent the number of complementary predictions. The results indicate that CRYSTALP2 is complementary to both XtalPred and ParCrys. For example, results on the TEST-NEW dataset show that CRYSTALP2 provides correct predictions for about 14.8% and 12.8% of the input protein chains that XtalPred and ParCrys, respectively, predict incorrectly. At the same time, XtalPred and ParCrys provide correct predictions for 15.5% and 14.2% of chains from the TEST-NEW dataset, respectively, for which CRYSTALP2 makes mistakes. Overall, the predictions of CRYSTALP2, and XtalPred and ParCrys overlap for only 69.8% and 73% of the input chains, respectively. To further investigate the complementarity we implemented a majority vote based meta-classifier that takes predictions from CRYSTALP2, XtalPred and ParCrys for a chain, and outputs the classification that at least 2 out of 3 methods agree on. A meta-classifier will improve on the individual base classifiers if and only if the base classifiers are complementary. Our meta-classifier obtains an accuracy equal to 73.4% and MCC equal 0.48 on the TEST-NEW dataset. When compared with the best accuracy on this dataset obtained by ParCrys, the majority vote predictor reduces the error rate by (73.4 - 70.6)/(100 - 70.6) = 2.8/29.4 = 9.5%. We thus conclude the proposed CRYSTALP2 method, ParCrys and XtalPred are complementary to each other, provide comparable prediction quality, and outperform the other three methods. We emphasize that complementarity between CRYSTALP2 and XtalPred suggests that the computational black-box methods, such as CRYSTALP2, provide useful support for the "manual" work of structural biologists as modelled in XtalPred.

**Table 3:** Comparison of predictions generated by CRYSTALP2, XtalPred and ParCrys on the TEST, TEST-RL and TEST-NEW datasets.

	**XtalPred**						**ParCrys**	
	
	**TEST dataset**		**TEST-RL dataset**		**TEST-NEW dataset**		**TEST-NEW dataset**	
	
**CRYSTALP2**	correct prediction	incorrect prediction	correct prediction	incorrect prediction	correct prediction	incorrect prediction	correct prediction	incorrect prediction
correct prediction	91	18	49	11	1091	295	1130	256
incorrect prediction	23	12	17	9	310	304	283	331

The table provides a breakdown of the predictions into those that were correct for both CRYSTALP2 and XtalPred (or ParCrys), correct for CRYSTALP2 and incorrect for XtalPred (or ParCrys), incorrect for CRYSTALP2 and correct for XtalPred (or ParCrys), and incorrect for both CRYSTALP2 and XtalPred (or ParCrys).

### Discussion of the proposed sequence representation

The 88 features selected for CRYSTALP2 include elements of the composition and collocation vector, which are computed directly from the sequence, and pI and hydrophobicity, which are derived from the sequence by considering specific physicochemical properties of the amino acid chains. We note that the two latter features were used in several past studies [16,17,21,23], while the former set of 86 features is introduced in this work as an extension of work done in [22]. We investigate whether these two sources of data, i.e., sequence and physicochemical properties of the sequence, provide complementary or redundant information in the context of predicting crystallization propensity.

**Figure 2:**
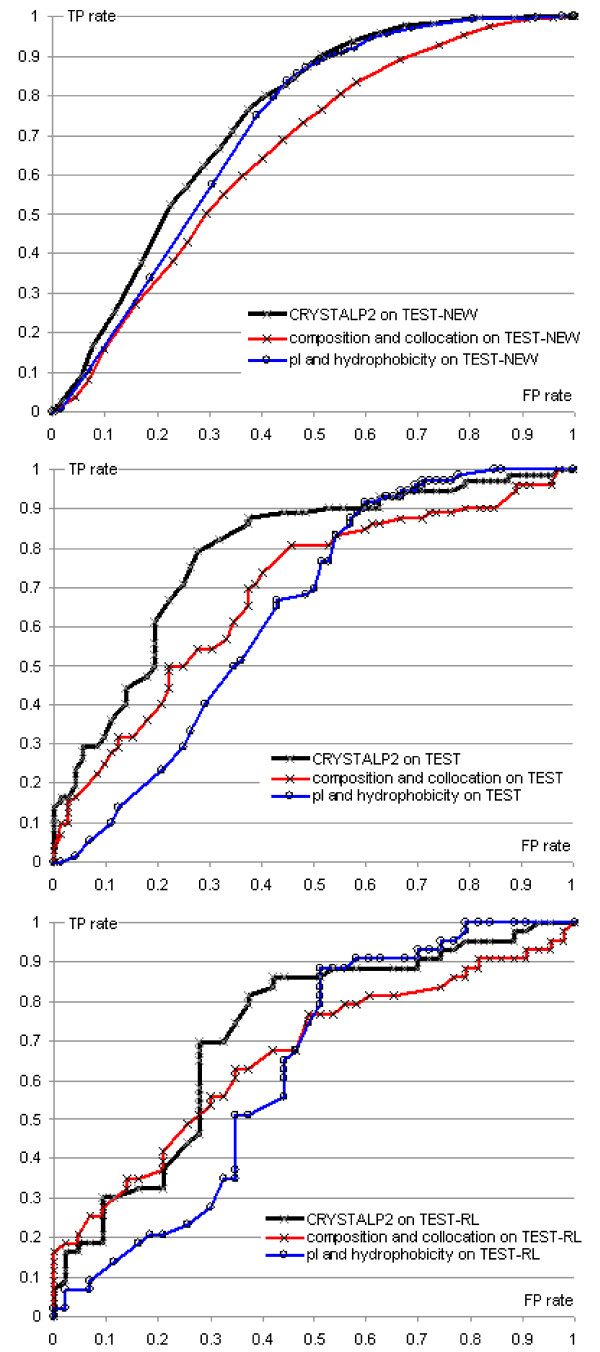
**ROC curves for CRYSTALP2, the predictions based on the 86 composition and collocation features, and a method that uses only pI and hydrophobicity features**. Top panel shows results on the TEST-NEW dataset, middle panel on the TEST dataset and bottom panel on the TEST-RL dataset.

Table 4 compares the quality of predictions when using three feature subsets: 1) pI and hydrophobicity; 2) composition and collocation-based features; 3) all 88 features. The experiments on the TEST-NEW, TEST, and TEST-RL datasets show that the usage of the combined set of 88 features results in superior predictions. The accuracy of CRYSTALP2 is improved by between 12.5% and 7.4%, depending on the dataset used, when compared to results obtained using only the 86 collocation-based features. Similarly, CRYSTALP2 predictions are improved by 0.5% to 9.7% when compared to predictions that utilize only the pI and hydrophobicity features. The improvements in AROC range between 0.06 and 0.1, and between 0.03 and 0.13 when comparing CRYSTALP2 with predictions based solely on the collocation-based features and on pI and hydrophobicity, respectively. We further explore the above differences in prediction quality using the ROC curves obtained for the three sets of features on the TEST-NEW, TEST and the TEST-RL datasets, see Figure 2. The curves show that the predictions that use 86 sequence-based features are characterized by better or comparable quality for low FP rates of up to about 0.55, 0.5, and 0.2 for the TEST, TEST-RL, and TEST-NEW datasets, respectively. The resulting higher TP rates for such low FP rates correspond to predictions in which a low number of sequences are incorrectly predicted as able to crystallize, while a higher number of chains are correctly predicted as crystallizable. In contrast, the usage of the two physicochemical properties to perform predictions results in higher TP rates for high values of FP rates. Based on these observations, we conclude that the two sources of data provide complementary information. We also observe that the combination of all 88 features results in ROC curves that work well for the entire range of the FP rates.

**Table 4:** Comparison of prediction quality measured via accuracy, MCC and AROC between the proposed method that uses the set of 88 features (including composition, collocation, pI and hydrophobicity), a method that uses the 86 composition and collocation features, and a method that uses only pI and hydrophobicity features.

**Dataset**	**Method (# features)**	**Accuracy**	**MCC**	**AROC**
TEST-RL	only pI and hydrophobicity (2 features)	67.4	0.38	0.63
	only composition and collocation (86 features)	62.8	0.26	0.66
	CRYSTALP2 (88 features)	69.8	0.40	0.72

TEST	only pI and hydrophobicity (2 features)	66.0	0.37	0.66
	only composition and collocation (86 features)	63.2	0.26	0.69
	CRYSTALP2 (88 features)	75.7	0.52	0.79

TEST-NEW	only pI and hydrophobicity (2 features)	68.8	0.41	0.71
	only composition and collocation (86 features)	61.9	0.24	0.66
	CRYSTALP2 (88 features)	69.3	0.39	0.74

Results are based on training the classification model on FEAT dataset and testing on TEST-RL, TEST, and TEST-NEW datasets, respectively.

In the following we investigate individual features used by CRYSTALP2. We show that the features based on the collocation of residues involve amino acids types that are also utilized in the crystallization enhancing mutagenesis. We then discuss the association of the individual features with the prediction outcomes.

The surface entropy reduction approach, i.e. point-mutation-based replacement of solvent-exposed residues having high conformational entropy (e.g. Glu (E), Gln (Q), and Lys (K)), with residues having lower conformational entropy and higher potential to mediate crystal contacts (such as Ala (A), Tyr (Y), Thr (T), Ser (S), and His (H)) provides a viable strategy to minimize the loss of conformational entropy upon crystallization and renders crystallization thermodynamically favorable [46,47,37]. The sites for mutagenesis are usually chosen considering their proximity in the sequence [37,47,48], which conceptually resembles our collocation vector approach. At the same time, the ParCrys and XtalPred methods use the composition of several AA types without considering their proximity. The eight AA types involved in surface entropy reduction are likely to be indicative of proteins with low/high crystallization propensity, and they occur in 73% of the features used by CRYSTALP2. Since the combined abundance of these AAs in protein chains is about 41%, their higher occurrence rate in our feature set demonstrates that CRYSTALP2 implicitly applies information about conformational entropy. We note that ParCrys uses the composition of Ser (S), Gly (G), Cys (C), Phe (F), Tyr (Y), and Met (M) AAs. Only two of these AA types are associated with the residues that are suggested in crystallization enhancing mutagenesis, which further supports our claim of complementarity between CRYSTALP2 and ParCrys. Similarly, XtalPred analyzes the composition of Cys (C), Met (M), Trp (W), Tyr (Y), and Phe (F) AAs, and again among these amino acid types only Y appears in the context of the mutagenesis.

Since CRYSTALP2 uses a nonlinear, black-box model to represent the relation between all input features taken together and the prediction outcomes, it is not possible to directly use this model to determine the associations of individual features with a specific outcome. Instead, we computed the biserial correlation coefficients between individual features and the annotation of the corresponding protein chains (crystallizable vs. noncrystallizable) to quantify the strength of the associations. Overall, we observe that 75 features used by CRYSTALP2 are characterized by weak absolute correlation coefficient values (<0.1). While individually these features little useful information, the classification model exploits these individually weak correlations by combining information from multiple features. The remaining 13 features having higher coefficient values include (the correlation coefficients are shown in brackets) L-E (0.28), SS (0.25), L (0.20), T-S (0.16), GL (0.15), R-S (0.14), I----E (0.14), L---L (0.14), F--S (0.12), E----F (0.11), S----H (0.11), S-T-S (0.11) and pI (0.3). We observe that the above collocations include AA types which are complementary to the AA types utilized by XtalPred (C, F, M, W, and Y; only one AA type, F, is in common). The same is true when we consider ParCrys, which uses the composition of C, F, G, M, S, and Y (only F, G, and S, are in common).

**Figure 3:**
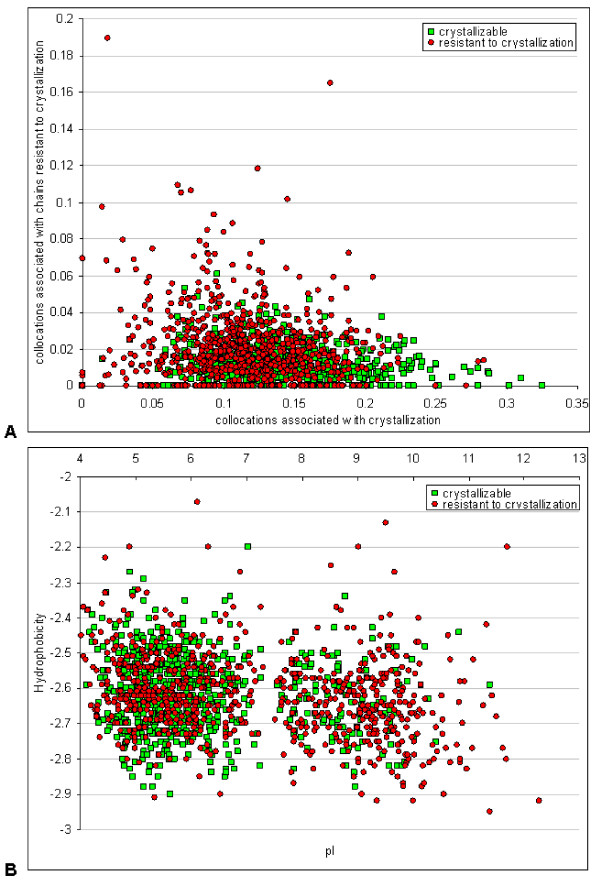
**The scatter plots showing the relation between the selected input features for crystallizable (denoted by green markers) and noncrystallizable (red markers) protein chains from the FEAT dataset**. Panel A shows relation between the summed values of the 6 collocations associated with crystallizable proteins (*x*-axis) and the 6 collocations associated with the noncrystallizable proteins (*y*-axis). Panel B shows relation between pI (*x*-axis) and hydrophobicity (*y*-axis).

In order to examine the relationship of the collocated peptides to crystallizability we divided the 13 features, excluding pI, into two subsets that can be expected to be associated with either the crystallizable or non-crystallizable class. Given that E, Q, and K are high entropy residues we considered collocations including these residues and residues associated with them (i.e., L-E, I----E, E----F, L, GL, and L---L), as associated with non-crystallizable chains. Similarly, A, Y, T, S, and H, which have higher potential to mediate crystal contacts were used to annotate the remaining correlated collocations (i.e., SS, T-S, R-S, F--S, S----H, and S-T-S) as associated with crystallizable chains. We aggregated (summed) the corresponding feature values for each subset and contrasted the resulting values between the two outcomes, see Figure 3A. We also compared these results to the graph representing the pI and hydrophobicity, see Figure 3B. We observe that neither the usage of the collocations nor the pI and hydrophobicity yields a clear separation between the two classes of protein chains. At the same time, as expected, the chains with high occurrence of the collocations associated with crystallizable chains tend to lead to successful crystallizations and vice versa; see the lower-right and upper-left corners of Figure 3A, respectively. In contrast, Figure 3B shows that although higher pI values are associated with a smaller likelihood of crystallization, the hydrophobicity does not show any clear trend.

### Analysis of CRYSTALP2 predictions

We additionally examine the results obtained by CRYSTALP2 in our second test (training on FEAT, testing on the TEST-NEW, TEST and TEST-RL datasets). Two questions are of interest: 1) could the prediction quality improve if the size of the FEAT dataset were increased (more crystallization reports would become available)? and 2) how does the proposed method performs for each of the prediction outcomes.

#### Impact of the size of the training dataset

**Figure 4:**
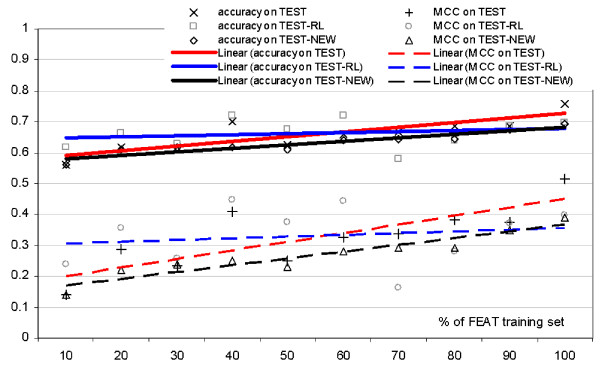
**Prediction quality (y-axis) measured with accuracy and MCC (shown using markers) when training the CRYSTALP2 model on subsets of the FEAT training dataset (x-axis) and testing on the TEST-NEW, TEST and TEST-RL datasets**. The solid and dashed lines show linear regression trends associated with the increasing size of the training dataset for the accuracy and MCC, respectively.

We select subsets of the FEAT dataset and re-train the NRBF classifier (using the same parameters) on these reduced datasets. The subsets are 10%, 20%, 30%,..., 90% of the FEAT dataset, selected randomly without replacement according to a uniform distribution. The prediction quality, measured by the accuracy and MCC, obtained for each subset of FEAT for the TEST-NEW, TEST-RL and TEST datasets is presented in Figure 4.

In TEST-RL, the prediction quality varies more substantially than for the TEST and TEST-NEW datasets. In spite of the above, we can discern a general upward trend in prediction quality for the three datasets. The trends for the TEST and TEST-NEW datasets are clearer and we observe that the prediction quality improves as more of the FEAT dataset is included in training, and reaches its maximum when the entire FEAT dataset is used. Most importantly, we observe that the rate of improvement is relatively constant, even when considering large fractions of the training dataset, i.e., 80, 90, and 100%. Interpolation of this trend suggests that inclusion of additional data in the training dataset could result in a further increase of the prediction quality.

The linear regressions in Figure 4 show that the improvements have larger magnitude for the TEST and TEST-NEW datasets than for the TEST-RL dataset, which highlights the difference between these datasets. We note that FEAT, TEST-NEW and TEST include sequences of unrestricted size, while TEST-RL only includes sequences between 46 and 200 residues in length. This difference in the distribution of the sequence sizes is a likely cause of the stronger improvements in the case of the TEST and TEST-NEW datasets.

#### Results for prediction of crystallizable and noncrystallizable proteins

We analyze the performance of the CRYSTALP2 method separately for the prediction of the crystallizable and noncrystallizable proteins. The prediction quality for each of the two outcomes is measured using sensitivity = TP/(TP + FN) and specificity = TN/(TN + FP), see Table 5. We note that these two measures are symmetric for two-class classifications, i.e., specificity of one class equals sensitivity of the other class and vice versa. The results on the TEST-NEW, TEST and TEST-RL are consistent and they show that CRYSTALP2 provides higher sensitivity for the prediction of the crystallizable proteins. This means that a higher fraction of the actual (true) crystallizable chains is correctly predicted when compared with the predictions for the noncrystallizable chains. The number of noncrystallizable chains that were predicted as crystallizable is higher than the number of crystallizable chains that were predicted as noncrystallizable, which indicates that the proposed method is better in the context of confirming that a given chain is suitable for crystallization when compared with the task of confirming that the crystallization fails.

**Table 5:** Comparison of prediction quality measured with sensitivity and specificity for the prediction of the crystallizable and noncrystallizable proteins by the CRYSTALP2 method.

	**crystallizable proteins**		**Noncrystallizable proteins**	
	
**Dataset**	**sensitivity**	**specificity**	**sensitivity**	**specificity**
TEST-RL	74.4	65.1	65.1	74.4
TEST	79.1	72.2	72.2	79.1
TEST-NEW	76.1	62.6	62.5	76.1

Results are based on training the classification model on FEAT dataset and testing on the TEST-RL, TEST, and TEST-NEW datasets, respectively.

## Conclusion

We introduce a novel algorithm, CRYSTALP2, that predicts the propensity of a given protein chain to generate diffraction-quality crystals via current structural biology techniques. Our results indicate that hydrophobicity, isoelectric point, and the frequency of certain collocated di- and tripeptides are important predictors of crystallization. We show that the collocation features provide a complementary source of information when compared with the hydrophobicity and isoelectric point. CRYSTALP2 associates AA collocations corresponding to clusters of residues having low conformational entropy and high potential to mediate crystal contacts with crystallizable proteins. Clusters of residues having high conformational entropy are associated with the non-crystallizable proteins. Such patterns could serve as potential crystallization markers.

Test on several independent datasets show that CRYSTALP2 outperforms several existing methods such as SECRET, CRYSTALP and the OB-Score, and provides comparable and complementary results to the ParCrys and XtalPred methods. The complementarity between CRYSTALP2 and XtalPred suggests that the proposed black-box method is a useful adjunct to the current manual techniques of structural biologists, which are modelled in XtalPred. Our results suggest that an increase of the size of the training set, which would be caused by the continuing protein crystallization efforts, may results in an increase of the prediction quality of the CRYSTALP2. We also show that the proposed method performs better in predicting crystallizable proteins when compared with predicting noncrystallizable proteins.

We note that our method and all competing approaches, i.e., SECRET, CRYSTALP, OB-Score, XtalPred and ParCrys, take into account only intra-molecular factors that are encoded in the protein chain. They may not provide reliable predictions when inter-molecular factors such as protein-protein and/or protein-precipitant interactions, buffer composition, precipitant diffusion method, gravity, etc. must be considered. All of these sequence-based predictors are limited to predicting crystallization propensity for non-redundant chains; they should not be used when assessing crystallization of homologues. In the latter case we recommend the use of the web server at [37]. Finally, our predictions concern only soluble proteins, as only such proteins were used to train and validate the prediction methods. In spite of these limitations, methods such as the proposed CRYSTALP2 should find useful applications. For instance, a potential application area is the Structural Genomics initiative where structures are sought for a protein that represents a given protein family rather than for a particular protein chain [8-11].

## Authors' contributions

LK contributed to the conception of the proposed method and the design of the feature sets and the classifier, helped in performing the tests, contributed to the evaluation and interpretation of the results, and wrote the manuscript. AR and SA contributed to the design of the feature sets and the classifier, computed the features, helped in performing the tests, and contributed to the evaluation of the results. SD helped in performing the tests and contributed to the evaluation of the results and to the writing of the manuscript. MM contributed to the experimental tests and the evaluation of the results. SJ contributed to the interpretation of the results and writing of the manuscript. All authors have read and approved the final version of the manuscript.
